# Comparison of pain intensity and impacts on oral health-related quality of life between orthodontic patients treated with clear aligners and fixed appliances: a systematic review and meta-analysis

**DOI:** 10.1186/s12903-023-03681-w

**Published:** 2023-11-24

**Authors:** Qiuying Li, Yugui Du, Kai Yang

**Affiliations:** Department of Orthodontics, School of Stomatology, Capital Medical University, No.4, Tiantanxili, Beijing, China

**Keywords:** Clear aligners, Fixed appliances, Oral health-related quality of life, Pain

## Abstract

**Objective:**

This study aimed to compare the pain intensity and impacts on oral health-related quality of life (OHRQoL) between orthodontic patients treated with clear aligners (CAs) and fixed appliances (FAs).

**Methods:**

A systematic search was conducted up to December 2022 using PubMed, Web of Science, Cochrane Central Register of Controlled Trials, and Embase. Randomized controlled trials (RCTs) and prospective non-randomized controlled trials (non-RCTs) comparing pain intensity or OHRQoL between patients treated with CAs and FAs were included. The risk of bias (RoB) of individual studies was evaluated using the Cochrane RoB tool 2.0 and ROBINS-I tool for RCTs and non-RCTs, respectively. Further, meta-analyses were separately conducted for each included study using the total oral health impact profile (OHIP)-14 and visual analog scale (VAS) scores to evaluate OHRQoL and pain intensity, respectively.

**Results:**

Overall, 12 studies (5 RCTs and 7 non-RCTs) were included in the study. Subgroup analyses conducted according to the total OHIP-14 scores revealed that patients treated with CAs had higher OHRQoL at 1 week, 1 month, and 6 months of the treatment. Meanwhile, subgroup analyses conducted according to the VAS scores revealed that pain levels were lower in the CA group only at 3 and 4 days of the treatment.

**Conclusions:**

Patients treated with clear aligners had higher OHRQoL than those treated with fixed appliances during orthodontic treatment. However, OHRQoL appeared to be similar between the two groups at the end of the treatment. Moreover, patients treated with clear aligners experienced lesser pain than those treated with fixed appliances on the third and fourth day after the initial treatment. The difference in pain intensity between the two treatment modalities was not noted at other time points.

**Supplementary Information:**

The online version contains supplementary material available at 10.1186/s12903-023-03681-w.

## Introduction

Malocclusion is known to have a negative effect on physical, social, and psychological well-being of patients [1]. Notably, an increasing number of patients have been seeking orthodontic treatment for various reasons, such as esthetic improvement, better oral function, and psychological well-being.

Modern orthodontic treatments are aimed at offering more comfortable experiences to these patients. However, during orthodontic treatment, pain and a decrease in oral health-related quality of life (OHRQoL) are inevitable, especially in the initial phase of the treatment [2, 3]. OHRQoL describes the patient-perceived impact of orofacial conditions and dental interventions. It is a comprehensive concept considerably influenced by various factors, such as physical health, psychological state, social relationships, and environment [4]. Thus, assessing the impact on OHRQoL may be extremely helpful for researchers and clinicians.

Recently, with the innovation in thermoplastic materials and computer technology advancements, clear aligners have become widespread. Furthermore, owing to esthetics, comfort, and easy oral hygiene maintenance, clear aligner therapy has been preferred by patients [5, 6]. Several studies have compared pain intensity and impacts on OHRQoL between patients treated with clear aligners and fixed appliances; however, their conclusions remain controversial. For example, Gao et al. reported that patients treated with clear aligners had lower pain levels and higher OHRQoL during the initial stage of orthodontic treatment [7]. Moreover, Shalish et al. used a validated OHRQoL questionnaire to compare pain perceptions and four areas of dysfunction between patients treated with clear aligners and fixed appliances. During the first week of treatment, they found no significant differences in pain levels, general activity disturbances, or oral dysfunction between the two groups [8]. Furthermore, Zhang et al. conducted a systematic review to summarize the effects of clear aligner therapy on OHRQoL; however, their review did not include high-quality prospective trials [9]. In addition, to the best of our knowledge, there is no meta-analysis on this topic to date.

Thus, the present systematic review and meta-analysis aimed to compare the pain intensity and impacts on OHRQoL between patients treated with clear aligners and fixed appliances in order to assist clinicians and patients in choosing the most appropriate treatment modality based on pain and OHRQoL parameters.

## Methods

### Protocol and registration

The protocol of the present study was registered in PROSPERO (https://www.crd.york.ac.uk/PROSPERO; registration number: CRD42023389320), and the study was conducted in accordance with the Preferred Reporting Items for Systematic Reviews and Meta-Analysis (PRISMA) 2020 statement [10].

### Eligibility criteria

The selection criteria for the studies to be analyzed were applied according to the PICOS (i.e., Population, Intervention, Comparison, Outcome, Study design) strategy as follows:

- *Population:* patients with permanent dentition requiring orthodontic treatment.
- *Intervention:* orthodontic treatment with clear aligners.
- *Comparison:* orthodontic treatment with fixed appliances.
- *Outcome:* the impact on patients’ oral health-related quality of life, as assessed by oral health impact profile (OHIP)-14 scores, was the primary outcome, with higher scores indicating lower OHRQoL; further, pain intensity, as assessed by visual analog scale (VAS) scores, was the secondary outcome.
- *Study design:* randomized controlled trial or prospective non-randomized controlled trial.

Conversely, animal studies; case reports or series; review articles; systematic reviews or meta-analyses; in vitro studies; retrospective studies; cross-sectional studies; and studies involving patients requiring orthognathic surgery or patients with poor oral health, systematic diseases, or physical or mental disabilities were excluded.

### Information sources and search strategy

The following electronic databases were systematically searched from their inception to December 2022: PubMed, Web of Science, Cochrane Central Register of Controlled Trials, and Embase. Notably, no filters were used for language, publication date, or methodology.

Representative keywords used for the search were as follows: “clear aligner”, “fixed appliances”, “oral health-related quality of life”, and “pain”. Search strategies for each database are listed in Additional file 1.

### Study selection

All studies were imported into EndNote 20 (Clarivate Analytics, Philadelphia, Pennsylvania, USA) for better selection. Subsequently, two authors of the present review (Li and Du) screened the titles and abstracts separately for selecting the relevant studies. Studies that could not be definitively excluded using the information obtained from the titles and abstracts were analyzed using full texts based on the eligibility criteria. Any disagreements between these two authors were resolved by discussion with the third author (Yang).

### Data extraction

Two authors (Li and Du) independently extracted data according to the PICOS strategy. Any discrepancies between the data extracted by them were discussed with the third author (Yang).

The following data were extracted from each study: name of first author, year of publication, country, study design, participant characteristics (sample size, gender, and age), intervention and comparison (type of appliance), treatment outcomes, timing of assessment, and author conclusions.

### Quality assessment

The quality of randomized controlled trials (RCTs) was assessed using the Cochrane Risk of Bias tool 2.0 [11], which comprised six domains: random sequence generation, allocation concealment, blinding of participants and personnel, blinding of outcome assessment, incomplete outcome data, and selective reporting. Conversely, the quality of non-randomized controlled trials was assessed using the ROBINS-I tool [12], which comprised seven domains: confounding, selection bias, bias in measurement classification of interventions, bias due to deviations from intended interventions, bias due to missing data, bias in measurement of outcomes, and bias in selection of the reported result. Finally, the identified risk of bias was classified as follows: “low risk”, “moderate risk”, “serious risk”, “critical risk”, and “no information”.

The overall quality of evidence was assessed using the Grades of Recommendations, Assessment, Development, and Evaluation (GRADE) system [13]. This assessment was made according to the following aspects: risk of bias, inconsistency, indirectness, imprecision, and other considerations. The overall quality of the evidence was rated as high, moderate, low, and very low.

### Data synthesis

A meta-analysis was conducted when ≥ 2 included studies reported the same outcomes using Review Manager 5.4 software (The Cochrane Collaboration, Copenhagen, Denmark). The intervention effect was expressed in terms of the mean difference (MD) and its 95% confidence interval (CI) if the results were obtained on the same scale. Further, *I*^2^ test was performed to evaluate heterogeneity. If* I*^2^ was < 50%, a fixed-effects model was used; alternatively, a random-effects model was used. If heterogeneity was too high (*I*^2^ > 50%), sensitivity and subgroup analyses were conducted. *P*-values of < 0.05 were considered statistically significant. Forest plots were created to illustrate the effect size and 95% CI of the intervention. Moreover, funnel plots were created to assess publication biases if meta-analyses included > 10 studies.

## Results

### Study selection

**Fig. 1:**
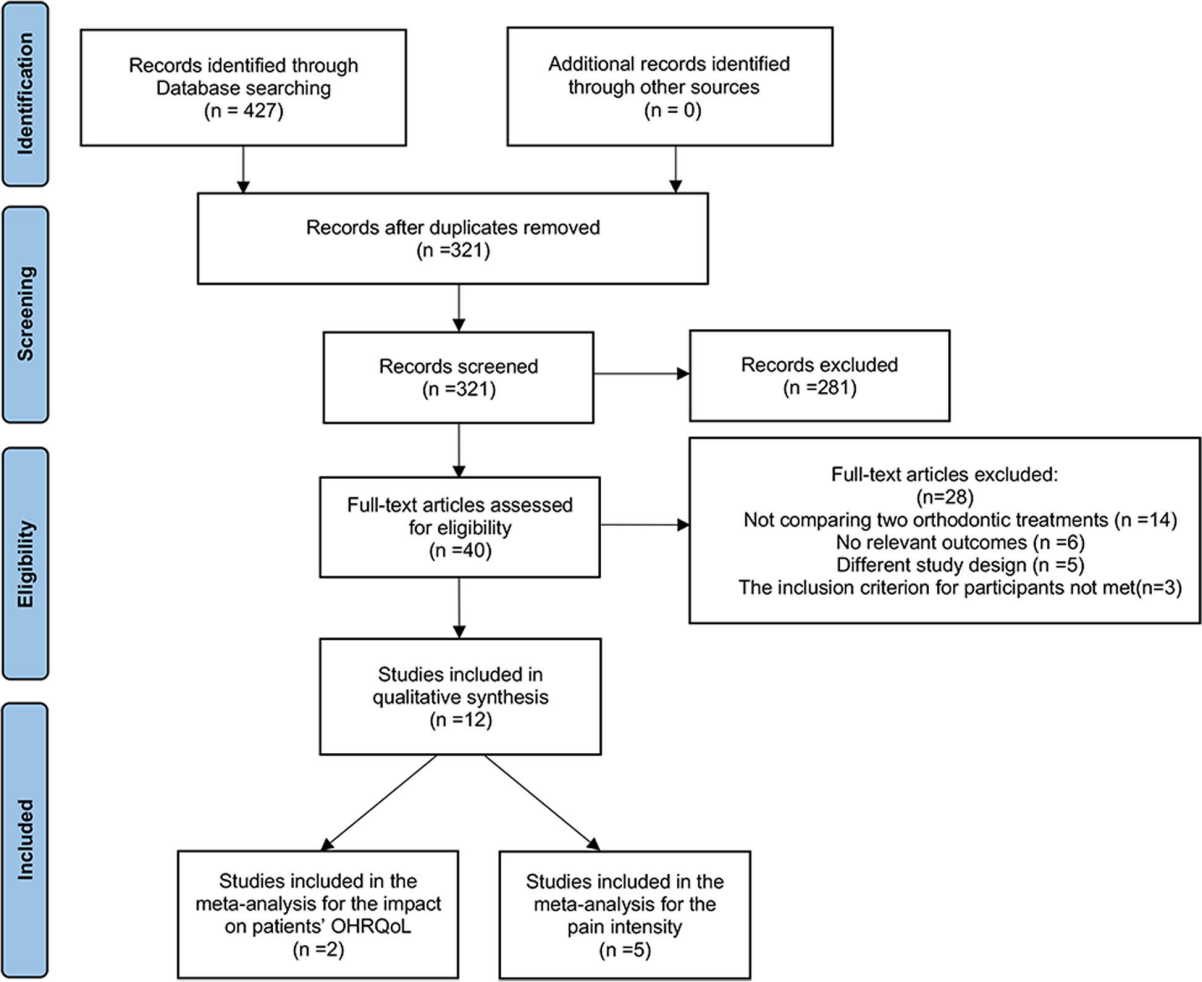
PRISMA flow diagram showing the literature selection process

Overall, 427 references were identified from the initial search. After removing duplicates, 321 articles were considered for screening. Following screening of the titles and abstracts of these articles, 40 articles remained for full-text evaluation. Further, based on the eligibility criteria, 12 studies were included in the qualitative synthesis [5, 7, 14–23]. Finally, two RCTs were included in the meta-analysis for the impact on patients’ OHRQoL [14, 21], and five studies were included in the meta-analysis for pain intensity [15–19]. Figure 1 presents the PRISMA flow diagram for literature selection progress.

### Study characteristics

Among all included studies, five were RCTs [14, 15, 19, 21, 23] and seven were prospective clinical studies [5, 7, 16–18, 20, 22]. Publication years of the included studies varied from 2007 to 2022, with most studies published after 2020 (8 of 12, 66.7%). Regarding geographic locations of the included studies, five [7, 14, 17, 21, 23], three [16, 18, 22], and four [5, 15, 19, 20] studies were conducted in Asia, Europe, and America, respectively.

The total number of patients enrolled in all studies was 955, including 40% men and 60% women. The mean age of participants in the clear aligner group was 21.27–38.0 years, whereas that of participants in the fixed appliance group was 20.75–33.8. Meanwhile, the age of participants was not mentioned in two studies [15, 23].

In 6 of 12 studies, the clear aligner group comprised patients who underwent treatment with Invisalign (Align Technology, CA, USA), whereas the clear aligner group of the remaining studies included patients who underwent treatment with other thermoformed clear aligners. Conventional buccal fixed appliances were provided to the fixed appliance group in 11 of 12 studies. Meanwhile, patients in four studies were provided other types of fixed appliances [17, 18, 22, 23]. The details of the study characteristics are provided in Table 1. Further details of the timing of assessment, specific outcomes of the individual studies, and author conclusions are provided in Additional file 2.

**Table 1:** Characteristics of the included studies

**First author (year)**	**Country**	**Study design**	**Sample size (male/female)**		**Age (mean ± SD)**		**Intervention**	**Comparison**	**Outcomes**
			**CA**	**FA**	**CA**	**FA**			
Alfawal (2022) [14]	Syria	RCT	22 (5/17)	22 (3/19)	25.40 ± 2.87	24.22 ± 2.99	Clear aligners	Conventional fixed appliances	OHIP-14 Treatment duration
Antonio-Zancajo (2020) [18]	Spain	Prospective clinical study	30 (16/14)	C:30 (13/17) LF:30 (12/18) L:30 (13/17)	33.4 ± 5.1	C:24.7 ± 4.1 LF:28 ± 9.7 L:33.8 ± 8.2	Invisalign®	Conventional brackets Conventional low-friction brackets Lingual brackets	OHIP-14 VAS
Gao (2021) [7]	China	Prospective cohort study	55 (13/42)	55 (13/42)	26.0 ± 5.47	24.6 ± 5.20	Clear aligners	Fixed appliances	VAS OHIP-14 S-AI
Jaber (2022) [21]	Syria	RCT	18 (9/9)	18 (8/10)	21.27 ± 1.87	20.86 ± 1.98	Clear aligners	Conventional fixed appliances	OHIP-14
Zamora-Martinez (2021) [22]	Spain	CCT	120 (61/59)		37.4 ± 14.6		Clear aligners	Fixed buccal mental brackets Fixed buccal esthetic/ceramic brackets Fixed lingual brackets	OHIP-14
Alcon (2021) [16]	Spain	Longitudinal clinical study	70 (33/37)	70 (35/35)	31.74 ± 11.39	26.97 ± 7.23	Clear aligners	Conventional fixed brackets	VAS
Almasoud (2018) [17]	Saudi Arabia	Prospective study	32 (10/22)	32 (12/20)	28.47 ± 8.17	23.56 ± 5.44	Invisalign®	Passive self-ligating fixed appliances	VAS
Casteluci (2021) [19]	Brazil	RCT	20 (13/7)	19 (12/7)	23.63 ± 5.62	20.75 ± 4.77	Invisalign®	Metallic fixed appliances	VAS Analgesic use
Fujiyama (2014) [20]	USA	CCT	38 (10/28)	EG:55 (20/35) EIG:52 (19/33)	26.64 ± 5.69	EG:26.45 ± 5.45 EIG:25.24 ± 6.51	Invisalign®	Conventional edgewise appliance	VAS
White (2017) [15]	USA	RCT	23 (11/12)	18 (6/12)	Not mentioned	Not mentioned	Invisalign®	Traditional fixed appliances	VAS Analgesic use Sleep disturbances
Diddige (2020) [23]	India	RCT	12 (6/6)	C:12 (6/6) SL:12 (6/6)	Not mentioned	Not mentioned	Clear aligners	Conventional fixed appliances Self-ligating fixed appliances	VAS Pain perception
Miller (2007) [5]	USA	Prospective longitudinal cohort study	33 (11/22)	27 (6/21)	38.0 ± 12.4	28.6 ± 8.7	Invisalign®	Fixed appliances	QoL impact score VAS Analgesic use

SD standard deviation, CA clear aligner, FA fixed appliance, RCT randomized controlled trials, CCT controlled clinical trial, OHIP-14 oral health impact profile 14, VAS visual analog scale, S-AI state anxiety inventory

### Quality assessment

**Fig. 2:**
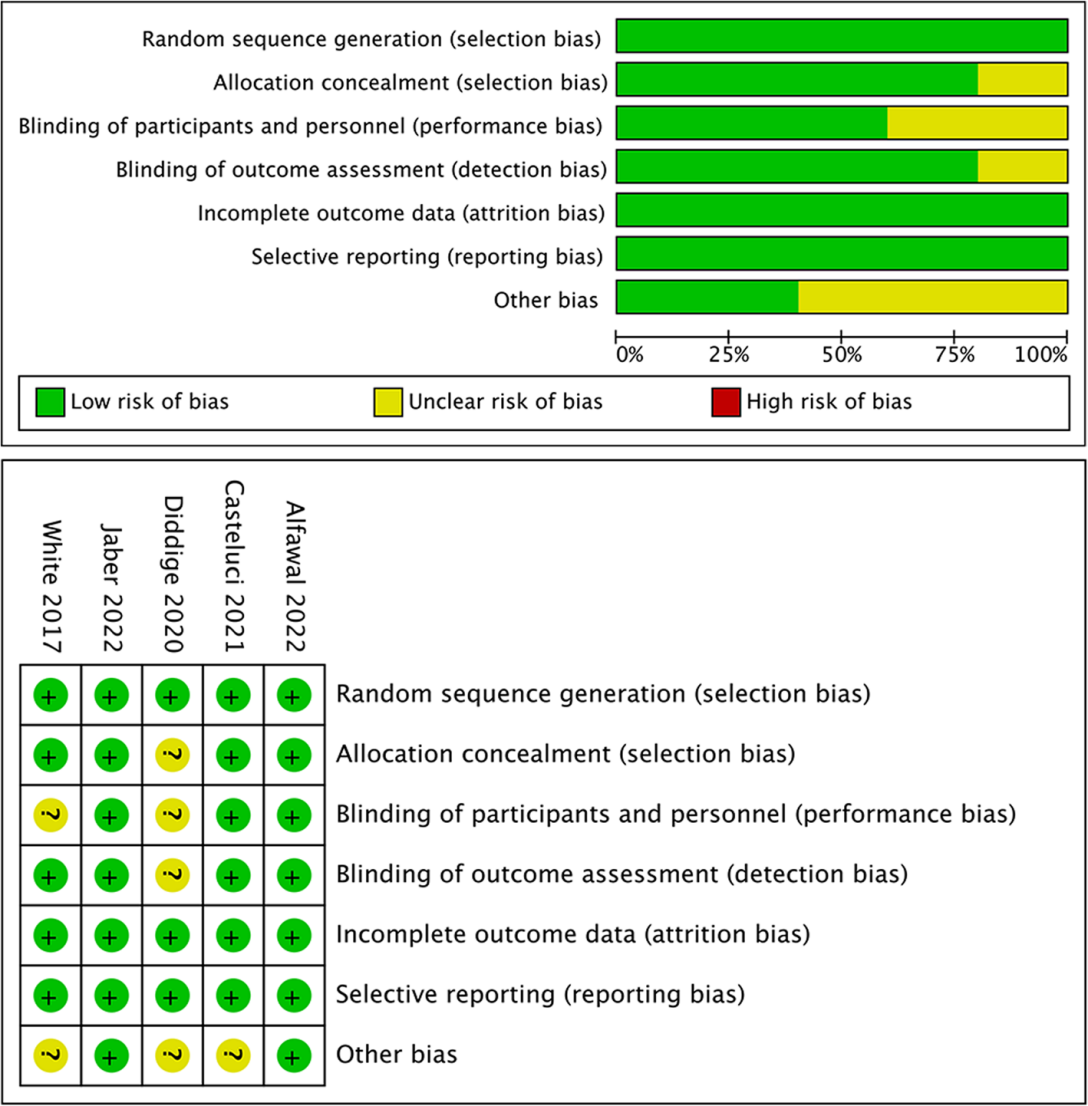
Risk of bias of included RCTs according to the RoB 2.0 tool

According to the Cochrane Risk of Bias tool 2.0, the overall risk of bias for RCTs ranged from low to moderate [14, 15, 19, 21, 23]. Notably, two studies were classified to have a low risk of bias [14, 21]. The details of the risk of bias for RCTs are presented in Fig. 2. The ROBINS-I tool was used to evaluate the risk of bias for seven non-RCTs [5, 7, 16–18, 20, 22]; only one study [7] had a low risk of bias, whereas five had a moderate risk [16–18, 22]. Furthermore, two studies were considered to have a serious risk of bias [5, 20]. Notably, the most affected domains were confounding bias and bias due to deviations from intended interventions. The results of the quality assessment are presented in Table 2.

**Table 2:** Risk of bias of included non-RCT according to the ROBINS-I tool

**Author (year)**	**Bias in/due to**							
	**Confounding**	**Selection of participants in the study**	**Measurement classification of interventions**	**Deviations from intended interventions**	**Missing data**	**Measurement of outcomes**	**Selection of the reported result**	**Overall**
Antonio-Zancajo (2020) [18]	Moderate	Low	Low	Low	No information	Moderate	Low	Moderate
Gao (2021) [7]	Low	Low	Low	Low	Low	Low	Low	Low
Zamora-Martinez (2021) [22]	Moderate	Low	Low	Low	No information	Low	Low	Moderate
Alcon (2021) [16]	Moderate	Low	Low	Moderate	No information	Low	Moderate	Moderate
Almasoud (2018) [17]	Low	Low	Low	Moderate	Low	Low	Low	Moderate
Fujiyama (2014) [20]	Serious	Moderate	Moderate	Moderate	Low	Low	Moderate	Serious
Miller (2007) [5]	Moderate	serious	serious	Moderate	Low	Low	Low	Serious

According to GRADE, the quality of evidence based on the total OHIP-14 scores was moderate (Additional file 3). Conversely, the quality of evidence based on the VAS scores was very low (Additional file 4). These low levels of evidence can be attributed to the limitations related to the study design (most included studies were observational) and inconsistencies.

### Results of the individual studies

Five studies compared the impact on OHRQoL between patients treated with clear aligners and fixed appliances [7, 14, 18, 21, 22]. They reported that orthodontic treatment adversely affected OHRQoL in both groups and that the OHRQoL of patients treated with clear aligners was higher than that of those treated with conventional fixed appliances during orthodontic treatment. According to three studies, the greatest reduction in OHRQoL occurred at the initial phase of the treatment and gradually improved in both groups [7, 14, 21]. Among these three studies, two reported that the total OHIP-14 scores peaked 1 week after the initial adjustment [14, 21], whereas the remaining one study reported that the scores peaked on the first day of the treatment [7]. Alfawal et al. and Zamora-Martinez et al. reported that OHRQoL was similar between the two groups at the end of the treatment [14, 22]. Regarding the seven domains of OHIP-14, Gao et al. [7] reported that after orthodontic treatment, the scores for all domains were significantly higher in the fixed appliance group than in the clear aligner group. Meanwhile, two studies reported that there were no significant differences in the scores for the functional limitation domain [14, 18]. Notably, two studies assessed fixed appliances other than conventional fixed appliances [18, 22]. Among these studies, one reported that patients treated with clear aligners had higher OHRQoL than those treated with any other types of fixed appliances [22], and the other reported that the total OHIP-14 scores of the lingual brackets group were lower than those of the Invisalign group, but there were no statistically significant differences between the two groups [18].

Nine studies used the VAS scores to compare pain intensity between the clear aligner and fixed appliances groups [5, 7, 15–20, 23]. These studies evaluated the pain level of patients during the first week of treatment. Among these studies, seven reported that the orthodontic pain level peaked on the first day after the initial adjustment and gradually decreased thereafter in both clear aligner and fixed appliance groups. Most studies reported that patients treated with clear aligners had lower pain levels than those treated with fixed appliances in the first week of treatment. However, Alcon et al. [16] reported that patients in the fixed appliance group had higher pain levels at 4 h (fixed appliance: 1.537 ± 1.220, clear aligner: 2.550 ± 2.390, *P* = 0.002), 8 h (fixed appliance: 3.231 ± 1.754, clear aligner: 3.424 ± 2.475, *P* = 0.606), and 7 days (fixed appliance: 0.425 ± 0.698, clear aligner: 0.629 ± 1.235, *P* = 0.241) of the treatment. Conversely, one study reported that the pain intensity between the two groups was not statistically significantly different at any time point (*P* > 0.05) [19]. Notably, three studies evaluated longer follow-up times. For example, in the study by White et al. [15], in addition to the first week following the initial adjustment, the first four days after the first and second month’s adjustment were evaluated. Further, White et al. concluded that the use of fixed appliances led to significantly more pain than the use of aligners at any time point. However, one study reported a different conclusion [16], wherein from the second month to the twelfth month of the treatment, patients with aligners experienced a higher level of pain. Casteluci et al. [19] found that the results for the first, third, and sixth months were the same as those for the first week. Three studies used different types of fixed appliances [17, 18, 23], and only one of them reported that the lingual brackets group had a lower level of pain than the aligner group; however, there were no statistically significant differences between the two groups (*P* > 0.05) [18].

### Synthesis of results

Meta-analyses were separately performed for each included study using the total OHIP-14 and VAS scores to assess OHRQoL and pain intensity, respectively. Further, a random-effects model was used for these outcomes. Given that the timing of the assessment could affect both OHRQoL and pain intensity, subgroup analyses were also performed. Remarkably, other confounding factors could also affect outcome assessment, but we did not perform subgroup analyses owing to the limited number of included studies.

#### Meta-analyses according to the total OHIP-14 scores

**Fig. 3:**
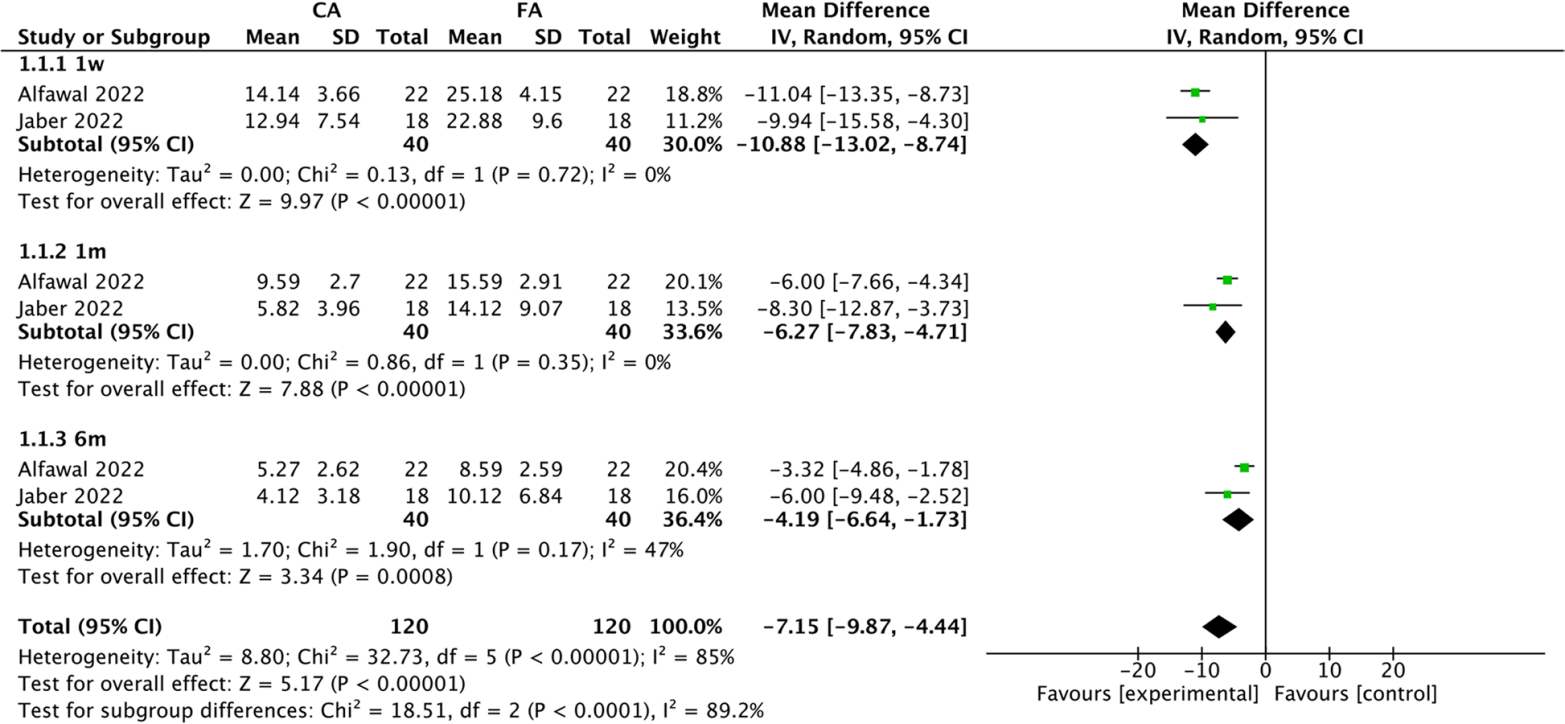
Forest plot of summary effect size (mean difference) in terms of the total OHIP-14 scores compared between the CA and FA groups at 1 week, 1 month, and 6 months. SD: standard deviation; IV: inverse variance; CI: confidence interval

Four studies reported the total OHIP-14 scores [14, 18, 21, 24]. For these scores, subgroup analyses were performed at the following time points: 1 week, 1 month, and 6 months. Two studies were excluded by sensitivity analyses owing to the high level of heterogeneity, but the overall effects for each subgroup remained the same [18, 22]. At 1 week, the pooled results indicated that patients treated with clear aligners had higher OHRQoL with no heterogeneity compared to those treated with fixed appliances (MD: − 10.88, 95% CI: [− 13.02, − 8.74], *P* < 0.00001; I^2^ = 0%,* P* = 0.72) [14, 21]. Similar results were found for 1 month (MD: − 6.27, 95% CI: [− 7.83, − 4.71], *P* < 0.00001; I^2^ = 0%, *P* = 0.35) [14, 18, 21] and 6 months (MD: − 4.19, 95% CI: [− 6.64, − 1.73], *P* < 0.00001; I^2^ = 47%, *P* = 0.17) [14, 21, 22] (Fig. 3).

#### Meta-analyses according to the VAS scores

**Fig. 4:**
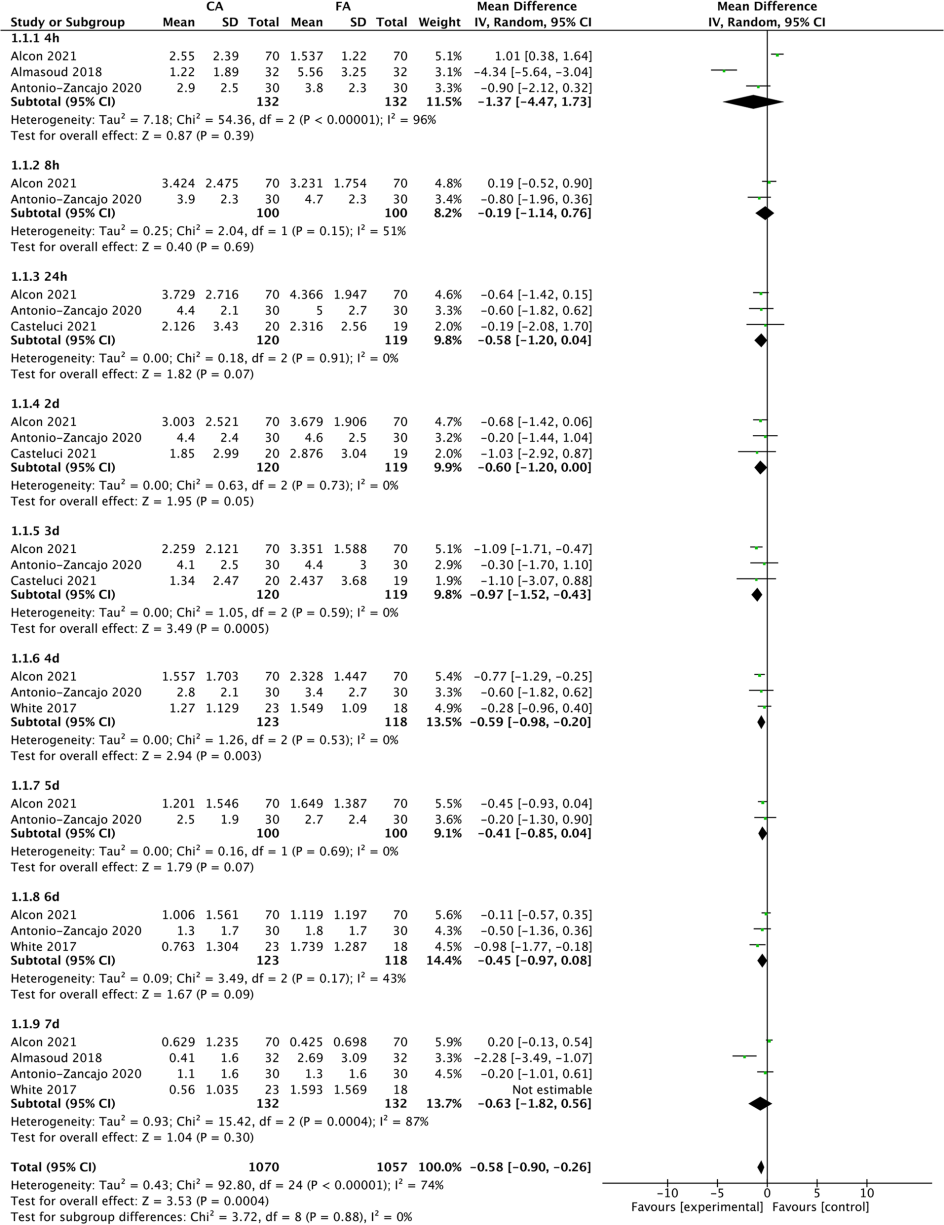
Forest plot of summary effect size (mean difference) in terms of the VAS scores compared between the CA group and FA group at 4 h, 8 h, 24 h, 2 days, 3 days, 4 days, 5 days, 6 days, and 7 days. SD: standard deviation; IV: inverse variance; CI: confidence interval

Five studies reported VAS scores [15–19]. For these scores, subgroup analyses were performed at the following time points: 4 h, 8 h, 24 h, and 2–7 days. One study was excluded by sensitivity analyses owing to the high level of heterogeneity at 24 h and 3 days, but the overall effects for each subgroup did not change [17]. At 4 h, 8 h, 24 h, 2 days, and 5–7 days, the treatment modalities did not affect pain intensity. At 3 days, the pooled results indicated that patients treated with clear aligners experienced lower pain levels with no heterogeneity compared to those treated with fixed appliances (MD: − 0.97, 95% CI: [− 1.52, − 0.43], *P* = 0.0005; I^2^ = 0%, *P* = 0.59) [16, 18, 19]. Similar results were found for 4 days (MD: − 0.59, 95% CI: [− 0.98, − 0.20], *P* = 0.003; I^2^ = 0%, *P* = 0.53) [15, 16, 18] (Fig. 4).

## Discussion

The measurement of OHRQoL has become an increasingly valuable assessment in dentistry given that patient-centered research helps reduce the gaps related to knowledge and perception between patients and clinicians and provides evidence that patients can understand [25]. A previous study reported that pain intensity is significantly related to oral health impact scores [26]. Accordingly, in the present study, we chose pain intensity and OHRQoL as the outcome indicators. Several tools have been designed and used to measure the impact on OHRQoL in the relevant literature [27–29]. Notably, OHIP-14 comprises seven domains: functional limitation, physical pain, psychological discomfort, physical disability, psychological disability, social disability, and handicap [30]. This tool has been widely used for patients undergoing orthodontic treatment owing to its high validity and reliability [31, 32]. In scientific research, VAS is the most commonly used tool for assessing pain intensity as it enables the use of parametric tests [33]. Notably, pain intensity is assessed using a VAS in the form of an unmarked 10-cm horizontal line with “no pain” at the left end and “severe pain” at the right end. For this assessment, patients are requested to mark the point on the line that best represents their pain severity. The scores on this scale range from 0 to 10 [17, 23]. Notably, to control methodological heterogeneity, we strictly limited the eligibility criteria to ensure that the effect values included OHIP-14 or VAS scores.

A previous systematic review reported that the differences between clear aligners and fixed appliances in terms of their effects on the overall OHRQoL are inconclusive [9]. However, based on the results of our study, patients treated with clear aligners had a lower overall OHIP-14 score, indicating a higher OHRQoL during orthodontic treatment. Regarding the seven domains assessed by OHIP-14, most studies reported that patients treated with clear aligners had lower scores in the domains of psychological discomfort, psychological disability, and physical disability [7, 14, 18, 21, 22]. In addition, given that clear aligners are more esthetic and invisible, it is not surprising that patients in the clear aligner group had fewer psychological problems than those in the FA group. Regarding physical disability, lower scores in the clear aligner group may be attributed to the fact that the aligners can be removed during meals without affecting chewing. However, differences in functional limitations between the two groups remain controversial. Some studies reported no significant differences between the two groups in terms of functional limitations such as difficulty in speaking or impaired sense of taste [14, 18]. Conversely, others reported that the clear aligner group had lower scores in terms of functional limitations [7, 21], which may be due to the smooth surfaces and reduced bulkiness of clear aligners, causing little irritation to the mucosa during various functional movements. In contrast, Alajmi et al. reported that patients treated with clear aligners presented significantly more limitations in the desired way of speaking and changes in speech delivery [34].

The present study reported that the pain levels in both groups peaked 24–48 h after the initial adjustment and gradually decreased thereafter during the initial phase of orthodontic treatment. Several studies have reported lower levels of pain in the clear aligner group during the first few days of treatment [5, 17, 20, 23]. However, based on our results, statistically significant differences were found only on the third and fourth days following the initial adjustment. Regarding the domain of longer evaluation time, the pain intensity between the two groups remains debatable [15, 16, 19].

Although we excluded one study by sensitivity analyses [17], the heterogeneity of some subgroups in the meta-analyses of pain intensity remained high. Meanwhile, several confounding factors between the two groups may have influenced the results of our study, such as patient- (age, sex, severity of malocclusion, and analgesic consumption) and intervention-related characteristics (types of appliances). Among the included studies, one study recruited adolescents [19]. Some previous studies reported that adolescents had lower pain levels than adults [35, 36]. Conversely, Johal A et al. reported that age did not affect the level of pain during orthodontic treatment [37]. It is known that compared with men, women prefer to choose more esthetic appliances (clear aligners). Diddige et al. reported that pain intensity was higher in women of all groups [23]. In contrast to this result, Miller et al. reported no significant differences in pain intensity between men and women [5]. Although there is no consensus on whether age or sex can affect pain intensity, they remain confounding factors that we cannot ignore. Notably, two of the nine included studies recruited patients with severe malocclusion who required extraction [7, 20], and patients undergoing extraction might have higher pain levels because of the surgical procedure. Furthermore, differences in the severity of malocclusion among participants of the included studies may affect the final results. In addition, owing to the material properties, clear aligners are not as efficient as fixed appliances in controlling complex tooth movements [38]. Thus, clear aligners are usually used for malocclusions of mild-to-moderate severity. In the future, well-designed studies are required to ensure that the severity of malocclusion is consistent between the study groups in order to eliminate the effect of this confounding factor. Another major confounding factor is the use of analgesics. Five included studies compared analgesic use between the two groups [5, 15, 17, 19, 23] and reported that a greater proportion of patients in the fixed appliance group used analgesics. One study found that patients in the fixed appliance group used analgesics more often than patients in the clear aligner group during the first week of activation [19]. The remaining included studies did not report on analgesic consumption. Notably, the use of analgesics may diminish the pain caused by orthodontic treatment itself. Moreover, the frequency, dosage, and type of analgesics used are major confounders that could not be neglected. Future studies are warranted to focus on this aspect. Previous studies investigated the pain intensity after bonding self-ligating and conventional brackets; however, their results remain inconclusive [39–41]. One study reported that the lingual brackets produced the most severe pain [8]. However, this outcome is contrary to that of the study by Antonio et al. who reported that patients with lingual brackets had the lowest level of pain [18]. In the present study, three articles using different types of brackets, except for conventional brackets such as self-ligating, lingual, ceramic, and low-friction brackets, were included. The above-mentioned confounding factors might affect the outcomes of our study if not well balanced. In addition, differences in the sequence of archwire replacement and the set of aligners may lead to heterogeneity [42].

The results of the present study should be interpreted with caution because of the following limitations. First, there was a lack of high-quality studies. Moreover, there were many confounding factors in the included studies, which might have led to heterogeneity. However, we could not perform subgroup analyses and assess publication biases due to the limited number of studies. Further well-designed long-term follow-up prospective clinical studies with large sample sizes and stringent methodological criteria are needed to achieve the highest level of evidence. These studies are required to better control confounding factors, such as age, sex, severity of malocclusion, type of appliance, and use of analgesics, between the study groups.

## Conclusions

The results of this systematic review and meta-analysis revealed that orthodontic treatment adversely affected OHRQoL. The greatest reduction in OHRQoL occurred at the initial phase of the treatment and gradually improved in both clear aligner and fixed appliance groups. Patients treated with clear aligners had higher OHRQoL than those treated with fixed appliances during orthodontic treatment. However, OHRQoL appeared to be similar between the two groups at the end of the treatment. Moreover, patients treated with clear aligners experienced lesser pain than those treated with fixed appliances on the third and fourth day after the initial treatment. The difference in pain intensity between the two treatment modalities was not noted at other time points.

Based on the low level of evidence, the results should be interpreted with caution. Future well-designed prospective clinical studies with large sample sizes and stringent methodological criteria are needed to be performed.

### Supplementary Information

**Additional file 1. **Database and search strategy.

**Additional file 2. **Details of included studies: time of evaluation, specific outcomes of individual studies, and authors’ conclusions.

**Additional file 3. **The quality of evidence based on GRADE for studies using the total OHIP-14 scores for the OHRQoL evaluation.

**Additional file 4. **The quality of evidence based on GRADE for studies using the VAS score for the pain intensity evaluation.

## Data Availability

All data generated or analyzed during this study are included in this published article (and its Additional files).

## List of abbreviations

OHRQoL: Oral health-related quality of life
CA: Clear aligner
FA: Fixed appliance
RCT: Randomized controlled trial
RoB: Risk of bias
ROBINS-I: Risk of bias in non-randomized studies-of interventions
OHIP: Oral health impact profile
VAS: Visual analog scale
PRISMA: Preferred reporting items for systematic review and meta-analysis
GRADE: Grade of recommendations, assessment, development, and evaluation
MD: Mean difference
CI: Confidence interval
