# Supplementary material for: Comparison of pain intensity and impacts on oral health-related quality of life between orthodontic patients treated with clear aligners and fixed appliances: a systematic review and meta-analysis
Source: BMC Oral Health. 2023 Nov 24;23:920. doi: 10.1186/s12903-023-03681-w (PMC10675971; doi:10.1186/s12903-023-03681-w)
Supplement: Supplementary file 1 — Additional file 1. Database and search strategy. [file 12903_2023_3681_MOESM1_ESM.docx]

**Additional file 1 Database and search strategy**

| Database | Search No. | Search strategy |
| --- | --- | --- |
| PubMed | 1 | "Orthodontic Appliances, Removable"[MeSH Terms] OR Removable Orthodontic Appliances [Title/Abstract] OR Removable Appliances [Title/Abstract] OR Invisible [Title/Abstract] OR aligner [Title/Abstract] OR Invisalign [Title/Abstract] OR Clear Aligner [Title/Abstract] OR Thermoplastic Orthodontic Appliances [Title/Abstract] |
|  | 2 | "Orthodontic Appliances, Fixed" [MeSH Terms] OR Fixed Orthodontic Appliances [Title/Abstract] OR Fixed Appliances [Title/Abstract] OR Braces [Title/Abstract] OR Brackets [Title/Abstract] |
|  | 3 | Quality of Life [Title/Abstract] OR QoL [Title/Abstract] OR Oral Health-Related Quality of Life"[Title/Abstract] OR OHRQoL [Title/Abstract] OR Satisfaction [Title/Abstract] OR Pain [Title/Abstract] OR Discomfort [Title/Abstract] |
|  | 4 | #1 AND #2 AND #3 |
| Web of Science | 1 | TS= (Orthodontic Appliances, Removable OR Removable Orthodontic Appliances OR Removable Appliances OR Invisible OR aligner OR Invisalign OR Clear Aligner OR Thermoplastic Orthodontic Appliances) |
|  | 2 | TS= (Orthodontic Appliances, Fixed OR Fixed Orthodontic Appliances OR Fixed Appliances OR Braces OR Brackets) |
|  | 3 | TS= (Quality of Life OR QoL OR Oral Health-Related Quality of Life OR OHRQoL OR Satisfaction OR Pain OR Discomfort) |
|  | 4 | #1 AND #2 AND #3 |
| The Cochrane Library | 1 | (Orthodontic Appliances, Removable OR Removable Orthodontic Appliances OR Removable Appliances OR Invisible OR aligner OR Invisalign OR Clear Aligner OR Thermoplastic Orthodontic Appliances): ti, ab, kw (Word variations have been searched) |
|  | 2 | (Orthodontic Appliances, Fixed OR Fixed Orthodontic Appliances OR Fixed Appliances OR Braces OR Brackets): ti, ab, kw (Word variations have been searched) |
|  | 3 | (Quality of Life OR QoL OR Oral Health-Related Quality of Life OR OHRQoL OR Satisfaction OR Pain OR Discomfort): ti, ab, kw (Word variations have been searched) |
|  | 4 | #1 AND #2 AND #3 |
| Embase | 1 | 'orthodontic appliances, removable': ab, ti OR 'removable orthodontic appliances': ab, ti OR 'removable appliances': ab, ti OR invisible: ab, ti OR aligner: ab, ti OR invisalign: ab, ti OR 'clear aligner': ab, ti OR 'orthodontic aligner': ab, ti OR 'thermoplastic orthodontic appliances': ab, ti |
|  | 2 | 'orthodontic appliances, fixed': ab, ti OR 'fixed orthodontic appliances': ab, ti OR 'fixed appliances': ab, ti OR braces: ab, ti OR brackets: ab, ti |
|  | 3 | 'quality of life': ab, ti OR qol: ab, ti OR 'oral health-related quality of life': ab, ti OR ohrqol: ab, ti OR satisfaction: ab, ti OR pain: ab, ti OR discomfort: ab, ti |
|  | 4 | #1 AND #2 AND #3 |
