# Supplementary material for: Comparison of pain intensity and impacts on oral health-related quality of life between orthodontic patients treated with clear aligners and fixed appliances: a systematic review and meta-analysis
Source: BMC Oral Health. 2023 Nov 24;23:920. doi: 10.1186/s12903-023-03681-w (PMC10675971; doi:10.1186/s12903-023-03681-w)
Supplement: Supplementary file 2 — Additional file 2. Details of included studies: time of evaluation, specific outcomes of individual studies, and authors’ conclusions. [file 12903_2023_3681_MOESM2_ESM.docx]

**Additional file2 Details of included studies: time of evaluation, specific outcomes of individual studies, and authors’ conclusions**

| First author (year) | Time of evaluation | Detailed outcomes (mean,SD)/(median,25-75 percentile) | | Conclusions |
| --- | --- | --- | --- | --- |
|  |  | CA | FA |  |
| Alfawal (2022) | pre-treatment(T0),1w(T1),1m(T2), 3m(T3),6m(T4), post-treatment(T5) | OHIP-14:  T0:12.5(10.75-15)  T1:14.14(3.66)  T2:9.59(2.70)  T3:8.18(3.17)  T4:5.27(2.62)  T5:0(0-1) | OHIP-14:  T0:12.5(11.75,14.25)  T1:25.18(4.15)  T2:15.59(2.91)  T3:11.46(2.63)  T4:8.59(2.59)  T5:1(0-1.25) | Patients treated with clear aligners reported higher OHRQoL and shorter treatment duration as compared to those treated with fixed appliances. |
| Antonio-Zancajo (2020) | OHIP-14: 1m  VAS:4h(T4h), 8h(T8h), 24h(T1), 2d-7d(T2-T7), and from the 7^th^ day(T18) | OHIP-14:1.7(1.9)  VAS (cm):  T4h:2.9(2.5)  T8h:3.9(2.3)  T1:4.4(2.1)  T2:4.4(2.4)  T3:4.1(2.5)  T4:2.8(2.1)  T5:2.5(1.9)  T6:1.3(1.7)  T7:1.1(1.6) | OHIP-14:3.8(2.1)-C; 4.5(2.8)-LF; 1.3(1.2)-L  VAS (cm):  T4h:3.8(2.3)-C; 2.0(1.9)-LF; 1.7(2.3)-L  T8h:4.7(2.3)-C; 3.4(2.2)-LF; 3.3(1.9)-L  T1:5.0(2.7)-C; 5.6(2.0)-LF; 4(1.9)-L  T2:4.6(2.5)-C; 5.8(1.9)-LF; 2.7(2.1)-L  T3:4.4(3.0)-C; 4.7(2.3)-LF; 1.5(1.7)-L  T4:3.4(2.7)-C; 4.2(2.4)-LF; 0.9(1.3)-L  T5: 2.7(2.4)-C; 3.0(2.5)-LF; 0.6(1.2)-L  T6: 1.8(1.9)-C; 2.5(2.2)-LF; 0.3(0.9)-L  T7: 1.3(1.6)-C; 1.6(2.1)-LF; 0.2(0.8)-L | There is a significant difference in pain and OHRQoL in orthodontic patients with conventional, low-friction, and lingual brackets and aligners. |
| Gao (2021) | VAS:1-14d  OHIP-14:1, 7, 14d  S-AI:1, 3, 5, 7, 14d | Not been reported | Not been reported | Patients treated with clear aligners experienced lower pain levels, less anxiety, and higher OHRQoL |
| Jaber (2022) | Pre-treatment (T0), 1w(T1), 2w(T2), 1m(T3), 6m(T4), 12m(T5) | OHIP-14:  T0:7.82(4.35)  T1:12.94(7.54)  T2:7.71(5.68)  T3:5.82(3.96)  T4:4.12(3.18)  T5:2.88(2.57) | OHIP-14:  T0:9.94(7.22)  T1:22.88(9.60)  T2:16.41(9.27)  T3:14.12(9.07)  T4:10.12(6.84)  T5:6.88(3.81) | Patients' treatment with clear aligners has less impact on OHRQoL than those treated using conventional fixed appliances during the first year of treatment. |
| Zamora-Martinez (2021) | pre-treatment(T0), 6m(T1), post-treatment(T2) | OHIP-14:  T0:13.5(10-25)  T1:15.0(10-21)  T2:3.0(1-6) | OHIP-14:  T0:11.5(7-17)-M;13.0(8-22)-E;12.0(6-24)-L  T1:24.0(19-31)-M;25.0(18-30)-E;21.0(13-27)-L  T2:2.0(0-5)-M;2.0(1-6)-E;2.0(0-5)-L | Patients underwent a significant reduction in quality of life during treatment in comparison with their pre-treatment condition but showed significant improvements at the end of treatment. |
| Alcon (2021) | monthly at 4h(T1), 8h(T2), 24h(T3), 2-7d(T4-T9) during the first 12 months | VAS (cm):  1m:1.976(2.295)  T1:2.550(2.390)  T2:3.424(2.475)  T3:3.729(2.716)  T4:3.003(2.521)  T5:2.259(2.121)  T6:1.557(1.703)  T7:1.201(1.546)  T8:1.006(1.561)  T9:0.629(1.235)  2m:1.288(1.835)  3m:1.197(1.904)  4m:0.998(1.675)  5m:0.884(1.483)  6m:0.926(1.510)  7m:0.791(1.442)  8m:0.691(1.369)  9m:0.627(1.318)  10m:0.740(1.510)  11m:0.525(1.246)  12m:0.452(1.145) | VAS (cm):  1m:2.175(1.984)  T1:1.537(1.220)  T2:3.231(1.754)  T3:4.366(1.947)  T4:3.679(1.906)  T5:3.351(1.588)  T6:2.328(1.447)  T7:1.649(1.387)  T8:1.119(1.197)  T9:0.425(0.698)  2m:0.869(1.159)  3m:0.642(1.009)  4m:0.607(1.004)  5m:0.405(0.792)  6m:0.430(0.852)  7m:0.331(0.731)  8m:0.573(0.962)  9m:0.386(0.807)  10m:0.380(0.761)  11m:0.269(0.657)  12m:0.202(0.580) | In the first month of treatment, the patients with conventional fixed multibrackets reported the highest levels of pain compared to those with removable aligners. From the second month on, this trend changed. The patients with removable aligners reported the highest levels of pain. Therefore, the orthodontic system used influenced the perception of pain in patients. |
| Almasoud (2018) | 4h, 24h, 3d, 7d | VAS (cm):  4h:1.22(1.89)  24h:1.38(2.69)  3d:0.66(1.8)  7d:0.41(1.6) | VAS (cm):  4h:5.56(3.25)  24h:6.0(3.41)  3d:4.59(3.32)  7d:2.69(3.09) | During the first week of orthodontic treatment, patients treated with Invisalign aligners reported lower pain than did those treated with passive self-ligating fixed appliances. |
| Casteluci (2021) | Pre-treatment, seven first days after appliance placement (T1), and seven first days after each return, during the following months:1m(T2), 3m (T3), 6m (T4) | VAS (mm):  T1:1d:15.5(2.0-45.0)  2d:14.0(1.5-39.0)  3d:8.0(0.0-31.0)  4d:2.5(0.0-15.5)  5d:1.0(0.0-16.0)  6d:0.0(0.0-10.0)  7d:0.0(0.0-4.0) | VAS (mm):  T1:1d:21.0(8.0-40.0)  2d:23.0(12.0-50.0)  3d:15.0(5.0-51.0)  4d:14.0(3.0-29.0)  5d:12.0(0.0-49.0)  6d:9.0(0.0-41.0)  7d:7.0(0.0-30.0) | The pain intensity, usually mild, was not influenced by the appliance design, although different patterns of reported pain seem to occur between groups. |
| Fujiyama (2014) | 1. First stage:60 s, 6h, 12h, 1-7d after the appliance delivery  2. Second stage:60 s, 6h, 12h, 1-7d(3w)  3. Third stage:60 s, 6h, 12h, 1-7d(5w) | Not been reported | Not been reported | Invisalign may offer less pain compared to the edgewise appliance during the initial stages of treatment. |
| White (2017) | Immediately after initial adjustment, each day for 7 days, 4 days after subsequent adjustment (after 1/2 months) | VAS (mm):  Initial adjustment:  Baseline:11.87(3.3-17.1)  1d:13.59(7.1-36.9)  2d:13.08(8.3-36.7)  3d:10.68(4.6-39.2)  4d:11.31(6.1-20.4)  5d:5.70(0.0-11.2)  6d:5.54(0.2-16.7)  7d:3.19(0.0-13.1)  1m:  Baseline:6.37(1.07-36.53)  1d:5.05(0.33-29.27)  2d:4.19(0.17-23.46)  3d:3.56(0.00-16.58)  4d:2.40(0.00-17.58)  2m:  Baseline:7.50(5.55-24.66)  1d:9.21(4.38-24.58)  2d:11.05(3.16-23.99)  3d:6.60(1.35-16.02)  4d:4.32(1.17-18.87) | VAS (mm):  Initial adjustment:  Baseline:16.17(7.62-37.32)  1d:16.87(10.83-33.13)  2d:19.64(12.81-29.27)  3d:10.58(6.67-19.73)  4d:16.33(8.38-21.94)  5d:17.83(13.7-36.8)  6d:15.03(10.3-26.3)  7d:13.80(7.0-26.5)  1m:  Baseline:16.17(7.62-37.32)  1d:16.87(10.83-33.13)  2d:19.64(12.81-29.27)  3d:10.58(6.67-19.73)  4d:16.33(8.38-21.94)  2m:  Baseline:7.50(5.55-24.66)  1d:22.39(14.77-32.43)  2d:19.06(11.26-38.30)  3d:15.15(7.04-25.70)  4d:23.26(11.02-30.73) | Patients treated with traditional fixed appliances reported greater discomfort and consumed more analgesics than patients treated with aligners. |
| Diddige (2020) | 4h, 24h, 3d, 7d | VAS:  4h:2.67  24h:2.72  3d:1.27  7d:1.20 | VAS:  4h:5.17-C;3.76-SL  24h:5.53-C;3.82-SL  3d:3.25-C;2.33-SL  7d:2.49-C;1.65-SL | During the first week of orthodontic treatment, patients treated with clear aligners reported lower pain than those treated with conventional and self- ligating appliances. |
| Miller (2007) | pre-treatment, 1-7d | Not been reported | Not been reported | Adults treated with Invisalign aligners experienced less pain and fewer negative impacts on their lives during the first week of orthodontic treatment than did those treated with fixed appliances. |
