# Supplementary material for: Comparison of pain intensity and impacts on oral health-related quality of life between orthodontic patients treated with clear aligners and fixed appliances: a systematic review and meta-analysis
Source: BMC Oral Health. 2023 Nov 24;23:920. doi: 10.1186/s12903-023-03681-w (PMC10675971; doi:10.1186/s12903-023-03681-w)
Supplement: Supplementary file 3 — Additional file 3. The quality of evidence based on GRADE for studies using the total OHIP-14 scores for the OHRQoL evaluation. [file 12903_2023_3681_MOESM3_ESM.docx]

**Additional file 3** The quality of evidence based on GRADE for studies using the total OHIP-14 scores for the OHRQoL evaluation.

| **Certainty assessment** | | | | | | | **№ of patients** | | **Effect** | | **Certainty** | **Importance** |
| --- | --- | --- | --- | --- | --- | --- | --- | --- | --- | --- | --- | --- |
| **№ of studies** | **Study design** | **Risk of bias** | **Inconsistency** | **Indirectness** | **Imprecision** | **Other considerations** | **CA** | **FA** | **Relative (95% CI)** | **Absolute (95% CI)** |  |  |
| **OHIP-14 - 1w** | | | | | | | | | | | | |
| 2 | randomized trials | not serious | not serious | not serious | serious | none | 40 | 40 | - | MD **10.88 lower** (13.02 lower to 8.74 lower) | ⨁⨁⨁◯ Moderate |  |
| **OHIP-14 - 1m** | | | | | | | | | | | | |
| 2 | randomized trials | not serious | not serious | not serious | serious | none | 40 | 40 | - | MD **6.27 lower** (7.83 lower to 4.71 lower) | ⨁⨁⨁◯ Moderate |  |
| **OHIP-14 - 6m** | | | | | | | | | | | | |
| 2 | randomized trials | not serious | not serious | not serious | serious | none | 40 | 40 | - | MD **4.19 lower** (6.64 lower to 1.73 lower) | ⨁⨁⨁◯ Moderate |  |

MD: mean difference; CI: confidence interval; OHIP-14: oral health impact file 14; OHRQoL: oral health-related quality of life; CA: clear aligner; FA: fixed appliance.
