# Supplementary material for: Comparison of pain intensity and impacts on oral health-related quality of life between orthodontic patients treated with clear aligners and fixed appliances: a systematic review and meta-analysis
Source: BMC Oral Health. 2023 Nov 24;23:920. doi: 10.1186/s12903-023-03681-w (PMC10675971; doi:10.1186/s12903-023-03681-w)
Supplement: Supplementary file 4 — Additional file 4. The quality of evidence based on GRADE for studies using the VAS score for the pain intensity evaluation. [file 12903_2023_3681_MOESM4_ESM.docx]

**Additional file 4** The quality of evidence based on GRADE for studies using the VAS score for the pain intensity evaluation.

| **Certainty assessment** | | | | | | | **№ of patients** | | **Effect** | | **Certainty** | **Importance** |
| --- | --- | --- | --- | --- | --- | --- | --- | --- | --- | --- | --- | --- |
| **№ of studies** | **Study design** | **Risk of bias** | **Inconsistency** | **Indirectness** | **Imprecision** | **Other considerations** | **CA** | **FA** | **Relative (95% CI)** | **Absolute (95% CI)** |  |  |
| **VAS - 4h** | | | | | | | | | | | | |
| 3 | observational studies | serious | serious | not serious | serious | none | 132 | 132 | - | MD **1.37 lower** (4.47 lower to 1.73 higher) | ⨁◯◯◯ Very low |  |
| **VAS - 8h** | | | | | | | | | | | | |
| 2 | observational studies | Serious | Serious | not serious | not serious | none | 100 | 100 | - | MD **0.19 lower** (1.14 lower to 0.76 higher) | ⨁◯◯◯ Very low |  |
| **VAS - 24h** | | | | | | | | | | | | |
| 3 | observational studies | Serious | not serious | not serious | not serious | none | 120 | 119 | - | MD **0.58 lower** (1.2 lower to 0.04 higher) | ⨁◯◯◯ Very low |  |
| **VAS - 2d** | | | | | | | | | | | | |
| 3 | observational studies | Serious | not serious | not serious | not serious | none | 120 | 119 | - | MD **0.6 lower** (1.2 lower to 0 ) | ⨁◯◯◯ Very low |  |
| **VAS - 3d** | | | | | | | | | | | | |
| 3 | observational studies | Serious | not serious | not serious | not serious | none | 120 | 119 | - | MD **0.97 lower** (1.52 lower to 0.43 lower) | ⨁◯◯◯ Very low |  |
| **VAS - 4d** | | | | | | | | | | | | |
| 3 | observational studies | serious | not serious | not serious | not serious | none | 123 | 118 | - | MD **0.59 lower** (0.98 lower to 0.2 lower) | ⨁◯◯◯ Very low |  |
| **VAS - 5d** | | | | | | | | | | | | |
| 2 | observational studies | serious | not serious | not serious | not serious | none | 100 | 100 | - | MD **0.41 lower** (0.85 lower to 0.04 higher) | ⨁◯◯◯ Very low |  |
| **VAS - 6d** | | | | | | | | | | | | |
| 3 | observational studies | serious | not serious | not serious | not serious | none | 123 | 118 | - | MD **0.45 lower** (0.97 lower to 0.08 higher) | ⨁◯◯◯ Very low |  |
| **VAS - 7d** | | | | | | | | | | | | |
| 3 | observational studies | serious | serious | not serious | serious | none | 132 | 132 | - | MD **0.63 lower** (1.82 lower to 0.56 higher) | ⨁◯◯◯ Very low |  |

MD: mean difference; CI: confidence interval; VAS: visual analog scale; CA: clear aligner; FA: fixed appliance.
